# Supplementary material for: Palbociclib-Induced Cellular Senescence Is Modulated by the mTOR Complex 1 and Autophagy
Source: Int J Mol Sci. 2023 May 26;24(11):9284. doi: 10.3390/ijms24119284 (PMC10252531; doi:10.3390/ijms24119284)
Supplement: Supplementary file 1 [file ijms-24-09284-s001.zip › Table S1.pdf]

Table S1. **Genomic analysis of the AGS and MCF-7 cell line.**

**cBioPortal (for cancer genomics) of 2826EXAMPLES ANALYZED**			**Altered by 10%**	**Altered by 8%**	**Altered by 4%**	**Altered by 9%**	**Altered by 14%**	**Altered by 14%**	**Altered by 12%**
**STUDIO ID**	**ID EXAMPLE**	**PATIENT ID**	**CCND1**	**CDK4**	**CDK6**	**pRB1**	**MTOR**	**RICTOR**	**RAPTOR**
**Ccle_broad_2019**	STOMACH	AGS	No alteration	No alteration	No alteration	No alteration	No alteration	No alteration	No alteration
**Cellline_ccle_broad**	STOMACH	AGS	No alteration	No alteration	No alteration	No alteration	No alteration	No alteration	No alteration
**Ccle_broad_2019**	BREAST	MCF-7	No alteration	No alteration	No alteration	No alteration	No alteration	No alteration	No alteration
**Cellline_ccle_broad**	BREAST	MCF-7	No alteration	No alteration	No alteration	No alteration	No alteration	No alteration	No alteration

Data provided by the cBioPortal for cancer genomics. The data shown correspond to the search in the three databases available on the portal. Of a total of 2,826 cell types analyzed, only some of them have mutations in the genes evaluated. The AGS and MCF-7 cell lines do not have alterations in the interrogated genes.
