# Supplementary figures and images for: Palbociclib-Induced Cellular Senescence Is Modulated by the mTOR Complex 1 and Autophagy
Source: Int J Mol Sci. 2023 May 26;24(11):9284. doi: 10.3390/ijms24119284 (PMC10252531; doi:10.3390/ijms24119284)

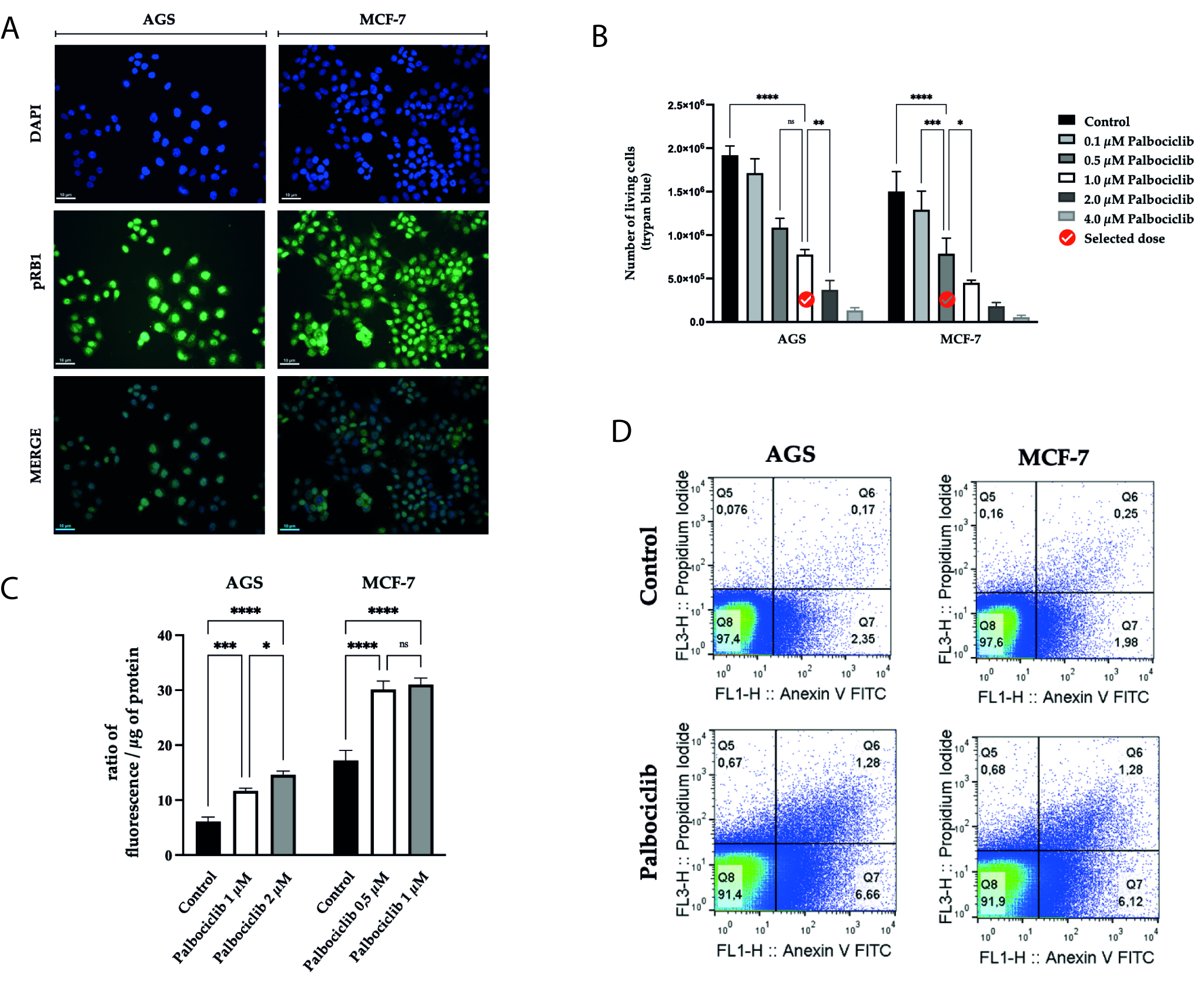

Supplement: Supplementary file 1 [file ijms-24-09284-s001.zip › Figure S1-AC.tif]

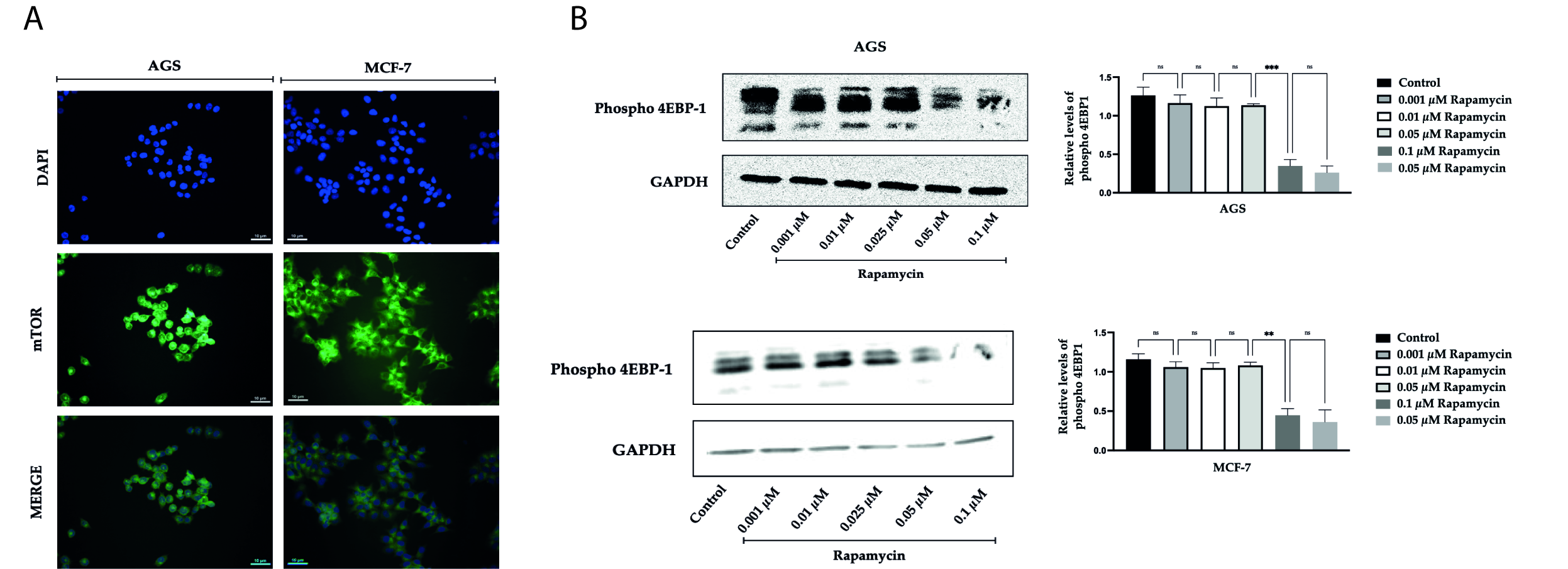

Supplement: Supplementary file 1 [file ijms-24-09284-s001.zip › Figure S2-AC.tif]

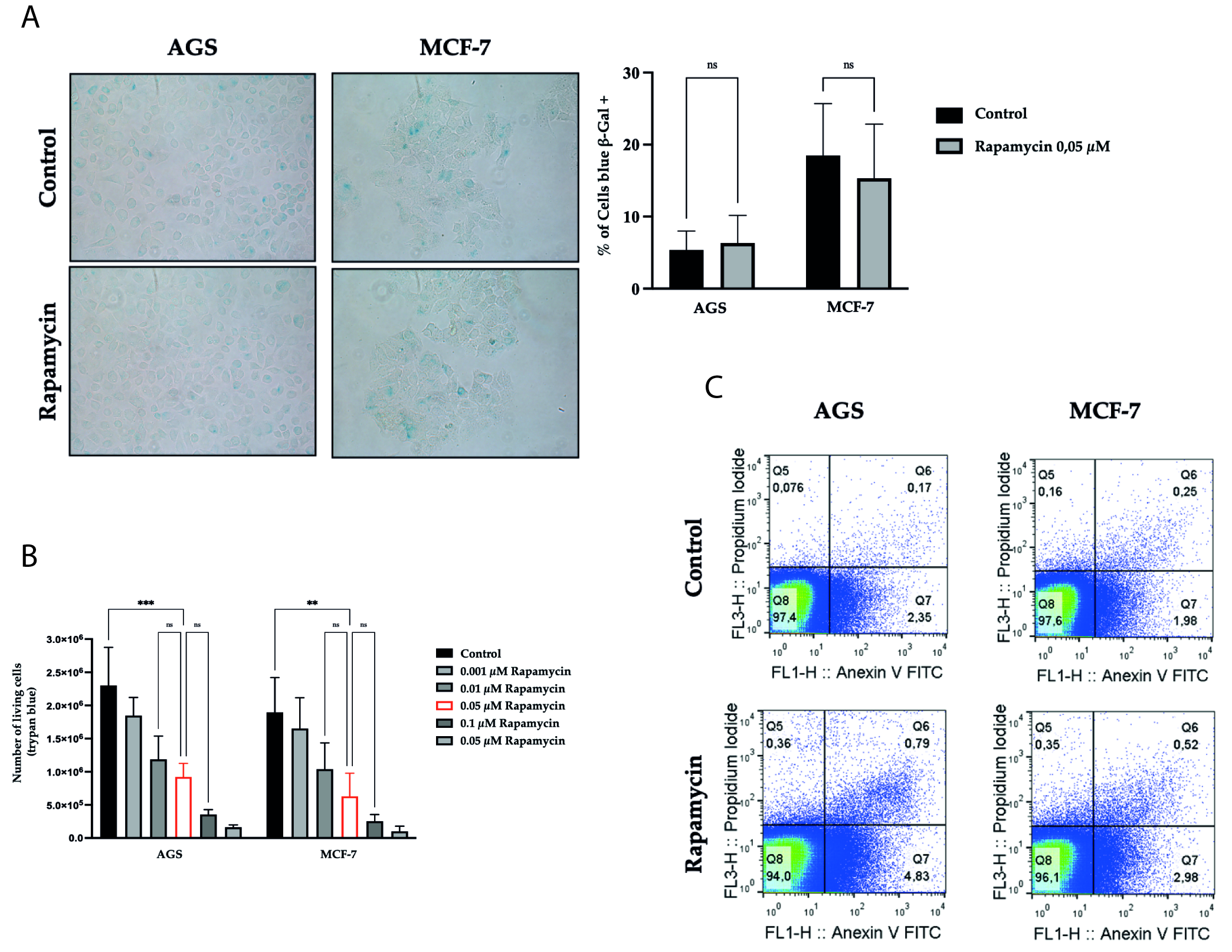

Supplement: Supplementary file 1 [file ijms-24-09284-s001.zip › Figure S3-AC.tif]

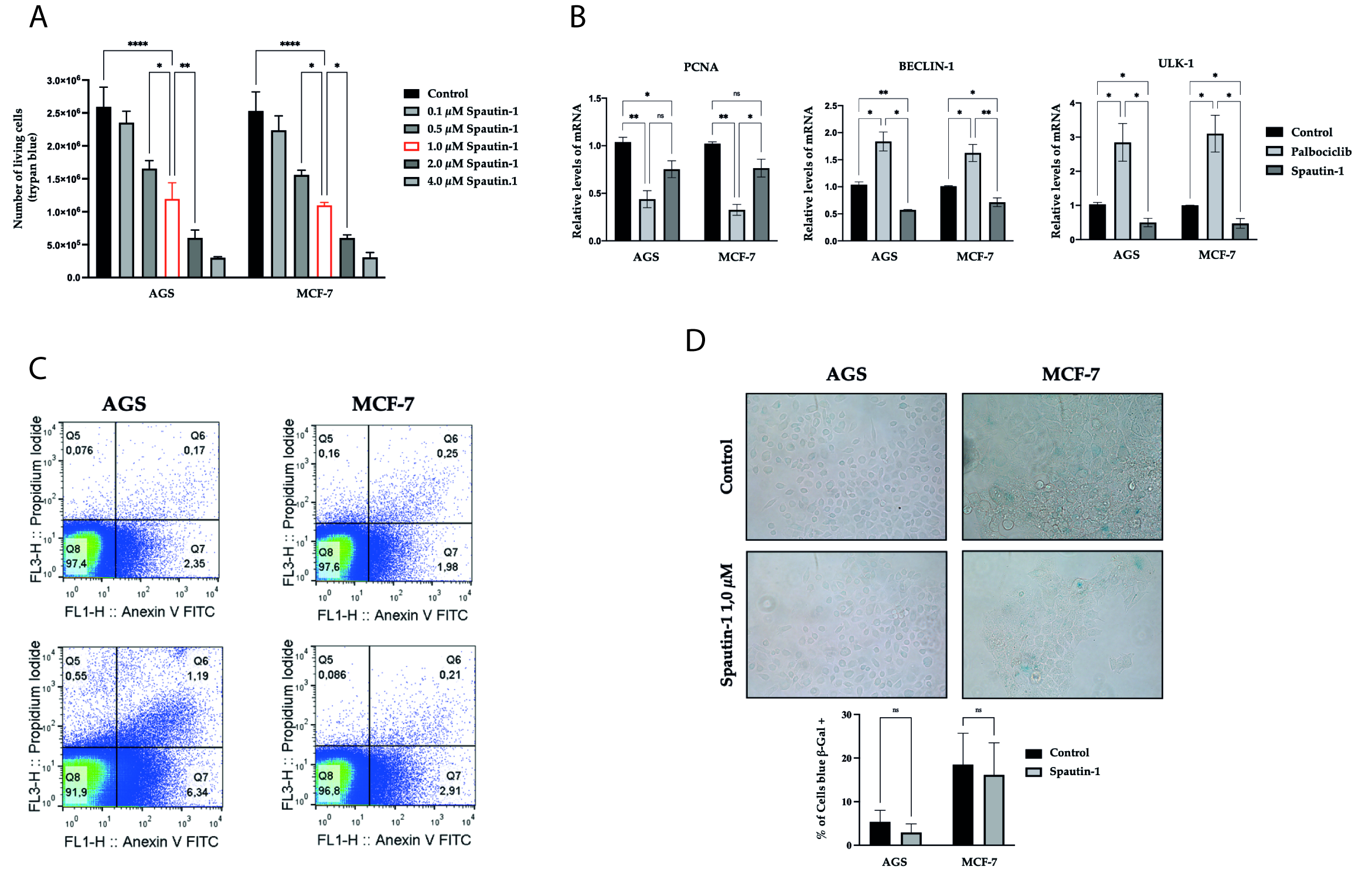

Supplement: Supplementary file 1 [file ijms-24-09284-s001.zip › Figure S4-AC.tif]
